# Sickness absence in pregnancy and sedentary behavior: a population-based cohort study from Norway

**DOI:** 10.1186/s12889-018-6379-4

**Published:** 2019-01-16

**Authors:** Idunn Brekke, Kåre Rønn Richardsen, Anne Karen Jenum

**Affiliations:** 1 Centre for Welfare and Labour Research - Norwegian Social Research, OsloMet - Oslo Metropolitan University, Post box 4 St. Olavs plass, N-0130 Oslo, Norway; 2 Faculty of Health Sciences - Department of Nursing and Health promotion, OsloMet – Oslo Metropolitan University, Post box 4 St. Olavs plass, N-0130 Oslo, Norway; 3 Faculty of Health Sciences - Department of Physiotherapy, OsloMet – Oslo Metropolitan University, Post box 4 St. Olavs plass, N-0130 Oslo, Norway; 4 General Practice Research Unit (AFE), Department of General Practice, University of Oslo, Institute of Health and Society, Post Box 1130 Blindern, 0318 Oslo, Norway

**Keywords:** Sickness absence, Sick leave, Sedentary time, Sedentary behaviour, Physical activity, Pregnancy, Maternal health

## Abstract

**Background:**

Sickness absence in pregnancy accounts for a large part of sickness-related absenteeism among women. Exercise in pregnancy is associated with a lower level of sickness absence, however little is known about how sedentary behaviour is related to sickness absence in pregnancy. In the current study, we hypothesize a positive association between sedentary hours/day and the risk of long-term sickness absence.

**Methods:**

Population-based cohort study of pregnant women attending three Child Health Clinics in Groruddalen, Oslo, 823 Women (74% of those eligible) were included between 2008 and 2010. Questionnaire data were collected at gestational weeks 10–20 (visit 1) and 28 (visit 2). Sedentary time and physical activity were objectively recorded at visit 1 with the multi-sensor SenseWear™ Pro3 Armband (SWA). Long-term sickness absence was self-reported at visit 2. We explored the association between sedentary time and long-term sickness absence in pregnancy using multiple logistic regression analysis.

**Results:**

The odds of long-term sickness absence was significantly increased per one-hour increase in daily sedentary time (odds ratio 1.45 [95% confidence interval 1.13–1.84]), providing support for our hypothesis that sedentary time is positively associated with long-term sickness absence.

**Conclusions:**

Pregnant women with a sedentary lifestyle have a higher risk of long-term sickness absence from work. Reducing sedentary time in pregnancy may improve health, and may, in turn reduce sickness absence in pregnancy.

## Background

Long-term sickness absence is a major public health problem and is very costly for the society. High rates of sickness absence among pregnant women are documented in several studies [1–6];it was 20.8% in Norway in 2014, and sickness absence in pregnancy accounts for a large part of sickness-related absenteeism in women [7]. The rates of sickness absence in pregnancy vary according to age, occupational class and immigrant background [8, 9]. Studies from several countries indicate that the amount of sickness absence has increased substantially in the last decades, and that there are no clear medical explanations for this development [2, 4, 10]. Common causes for sickness absence in pregnancy are back pain [6, 7], pelvic girdle pain [11], gestational diabetes [12], fatigue/sleeping problems and nausea [1]. However, absence could also be due to lack of adjustment of inappropriate working conditions [13, 14]. In addition to occupational exposures, worries, norms and attitudes [3, 15] are associated with sickness absence.

Moreover, evidence from a recent review including both intervention trials and observational studies suggests that physical activity is effective in reducing sickness absence in the general population [16]. Physical inactivity and sedentary behaviour are now considered among the main health risks globally [17]. Sedentary time refers to low-energy activities such as sitting and lying, while physical inactivity is defined as an insufficient physical activity level to meet present physical activity recommendations [18].

Increasing attention in the literature has been paid to the adverse health effects of a sedentary lifestyle [19]. Pregnant women spend the majority of their day as sedentary [20] and few meet the physical activity guidelines [21]. Sedentary time has a negative impact on several health outcomes [22, 23], and may impair pregnancy outcomes for both mother and child [24]. Moreover, studies indicate that the association between sedentary time and health outcomes is independent of physical activity levels [22, 23, 25]. Thus, people who meet the physical activity guideline, but remain predominantly sedentary, may experience the adverse health effects of sedentary behaviour [25]. It has been hypothesized that defects in metabolism, atherosclerosis, insulin resistance, and obesity mediate the adverse effects of sedentary behaviour on health [26]. Previous research has documented that exercise in pregnancy is associated with lower level of sickness absence [27]. Little is known about the association between sedentary behaviour and sickness absence. A study from The Netherlands [28] found no evidence for the link between sedentary behavior and sickness absence. However, the study did not examine this relationship in pregnancy and lacked objectively recorded data on sedentary time.

In the general population sedentary behaviour increases the risk of mortality [29] and many health problems such as type 2 diabetes [30], cardiovascular disease [31], metabolic syndrome [32] and low back pain [33]. Pregnant women spend more than half of their waking hours being sedentary [24] and are therefore at risk for developing health problems during pregnancy. The association between health and sickness absence is not constant, however previous research shows that poor health is associated with long-term sickness absence [34, 35]. Thus, health problems subsequent to a sedentary lifestyle during pregnancy are possible mediators in the relationship between sedentary behaviour and sickness absence in pregnancy. In contrast, health status prior to pregnancy could be a possible confounder affecting both sedentary time and sickness absence. Further, we have previously reported a strong association between pregnancy-induced emesis and long-term sickness absence [9] and emesis is also a plausible determinant of sedentary time and sickness-absence in pregnancy.

In the current study we aim to investigate if objectively recorded total sedentary hours/day during waking hours is associated with long-term sickness absence (i.e. > 15 weeks) in pregnancy. We hypothesize a positive association between sedentary hours/day and the risk of long-term sickness absence after adjusting for physical activity level and other potential confounding factors.

## Methods

This population-based cohort study was set up at three public Child Health Clinics located in three collaborating city districts in Groruddalen, Oslo, Norway. Groruddalen covers affluent as well as more deprived residential areas, and has a population with a diverse socioeconomic status. Antenatal care for uncomplicated pregnancies in Norway is mainly offered in primary care, and the majority of pregnant women residing in the three collaborating city districts (75–85%) attend the Child Health Clinics for antenatal care. From May 6 th 2008 to May 15th 2010, 823 women (74% of those eligible) were included [36]. All information material and questionnaires were translated to Arabic, English, Sorani, Somali, Tamil, Turkish, Urdu and Vietnamese, and quality-controlled by bilingual health professionals. Questionnaire data on health, pregnancy, physical activity, employment and demography were collected by project midwives during standardized interviews at gestational weeks 10–20 (visit 1) and 28 (visit 2). Women were eligible if they 1) lived in the districts, 2) planned to give birth at one of two study hospitals, 3) were less than 20 weeks pregnant, 4) could communicate in Norwegian or any of the other 8 languages available as translated questionnaires 5) were able to give a written consent to participate. Women with pre-gestational diabetes or other diseases necessitating intensive hospital follow-up during pregnancy, were excluded. In the present study, women were included provided that they attended in the study at 28 weeks of gestation and were employed 3 months prior to pregnancy. In total, 77% of the study population was employed 3 months prior to pregnancy.

### Ethical approval

Participants gave informed written consent before participation. The Norwegian Data Inspectorate and the Regional Committee for Medical and Health Research Ethics for South Eastern Norway approved the study protocol (2007/894).

## Measures

### Outcome variable

Doctor-certified sickness absence in pregnancy, was self-reported at study visit 2 (week 28) and recorded as number of weeks exceeding 2 weeks with full sickness absence. The outcome variable is measured as the total number of weeks with sickness absence in pregnancy until 28 weeks of gestation, and include both single and multiple spells. The outcome, long-term sickness absence, was treated as a binary variable in analyses, taking the value 1 if the it exceeded 15 weeks and 0 otherwise. The cut off was set at > 15 weeks which means that the women spend almost half of the time as pregnant on sick leave.

### Objectively recorded physical activity and sedentary time

The exposure variable sedentary hours/day and moderate-to-vigorous intensity physical activity (MVPA) minutes/day, were objectively recorded at visit 1 with the multi-sensor SenseWear™ Pro3 Armband (SWA) (BodyMedia Inc., Pittsburgh, Pennsylvania, USA) [37]. Women were asked to wear the SWA across the right triceps brachii over 4–7 days, and remove it only for water activities. We downloaded raw data integrated into 60-s epochs using the manufacturer’s software (SenseWear™ Professional Research Software Version 6.1, BodyMedia Inc.). Energy expenditure data was sampled at one-minute intervals, and sedentary time was defined as minute epochs with energy expenditure at 1.0–1.5 Metabolic equivalents (METs) (1 MET = 3.5 ml O^2^∙ kg^− 1^∙min^− 1^). In analysis sedentary time was expressed as hours/day, restricted to waking hours (i.e. 06:00–23:59), MVPA minutes/day was restricted to MVPA accumulated in bouts ≥10 min at ≥3 METs. MVPA minutes in bouts were extracted with SQL Server Management Studio (Microsoft®) and SQL Server Express version 11.0.5058.0 (Microsoft®). A valid SWA day was defined as ≥19.2 h of SWA wear time. Data on MVPA and sedentary hours/day were eligible if ≥3 valid SWA days were recorded [38].

## Confounders

### Health variables

All potential confounders originated from the interviews conducted at visit 1. Self-reported health three months pre-pregnancy was rated according to the response options 1) poor, 2) not good, 3) good or 4) excellent. In analyses, we collapsed poor and not good into one category. Self-reported pelvic girdle pain referred to pain over the pubic bone and either sacroiliac joint, and it was rated as 1) no pain, 2) some pain or 3) much pain. In analysis, we treated pelvic girdle syndrome as a binary variable; 1) No pain, or 2) pain (some pain or much pain at all three locations) [39].

### Sociodemographic variables

*Immigrant background* was categorized as follows: 0) majority women 1) immigrant women. Majority women were treated as the reference category, defined as being born in Norway with a Norwegian born mother. Age, number of children below 5 years in the household, highest completed education and percentage of full-time post (10–100, 100% = 37.5 h/week) were treated as continuous variables in the analysis. Occupation is in accordance with the occupation standard coding ISCO-08 [36]. *Occupational group* was categorized into 1) managers, professionals, 2) occupations that require a college education, 3) health and service workers, agriculture and industry 4) elementary occupations. The first category was treated as the reference category.

## Statistical methods

Descriptive analyses are presented with means (SD) and proportions (%).

The analyses of the association between sedentary hours/day and long-term sickness absence in pregnancy were performed using multiple logistic regression analyses to obtain odds ratios (ORs) and 95% confidence intervals (CIs). First, we performed univariate logistic regression analysis. Only significant variables were included in the multiple logistic regression analysis. We built three separate models to obtain estimates were physical activity and sedentary time were mutually adjusted (model 1), adjusted estimates after control for demographic and socioeconomic background variables (model 2), and, additionally, to control for health status pre-pregnancy and in early pregnancy (model 3). Statistical analysis was performed using STATA® 13. Statistical significance level was set to *p* < 0.05.

## Results

In total, 9% of the sample reported long-term sickness absence at visit 2 (Table 1). At visit 1, age ranged from 19 to 42 years, the mean MVPA was 24.7 min/day and the mean sedentary time was 12.1 h/day. Approximately 10% of the sample had pelvic girdle pain and 15% reported hyperemesis.

**Table 1:** Characteristics of study population, presented as mean (SD) and proportion

	mean (SD) and proportion
	*n* = 455
Sedentary time (ST) hour/day	12.1 (1.7)
moderate-to-vigorous intensity physical activity (MVPA) minute/day	24.7 (28.1)
Age (number of years)	29.7 (4.5)
Education (number of years)	13.9 (2.9)
Immigrant women (%)	48
Number of children < 5 years	0.5 (0.63)
Occupations that require a college education (%)	18
Health and service workers and industry (%)	29
Elementary occupations (%)	44
Managers/professionals (%)	9
Health status 3 months prior to pregnancy	
Excellent (%)	42
Good (%)	49
Poor/not good(%)	9
Pelvic girdle pain (%)	10
Hyperemesis (%)	15
Sickness absence (> 15 weeks) %	9

The relationship between long-term sickness absence and sedentary hours/day, is presented in three different models (model 1–3, Table 2). Across models, sedentary hours/day in early pregnancy was positively associated with higher odds of long-term sickness absence (Table 2). In the first model, the odds of long term sickness absence increased by 31% for each one hour-increase in sedentary time (OR 1.31, 95% CI: 1.05–1.62). The association was observed after adjustment for MVPA, while MVPA was not associated with long-term sickness absence in pregnancy (model 1). After adjusting for demographic and socioeconomic variables (model 2), sedentary time remained significantly associated with long-term sickness absence and the odds ratio increased slightly (OR 1.45, 95% CI: 1.13–1.84), while MVPA remained non-significant. The odds of having long-term sickness absence in pregnancy was higher for women in elementary occupations (OR 9.18, 95% CI: 1.22–69.34) than in occupations such as managers and professionals.

**Table 2:** Adjusted logistic regression (model 1–3) with regard to the impact of sedentary time on sickness absence > 15 weeks in pregnancy

n = observations	Model, OR (95% Cl)		
	Model 1 n = 455	Model 2 n = 455	Model 3 *n* = 438
Exposure variable			
Sedentary time (ST) hour/day	**1.31 (1.05–1.62)**	**1.45 (1.13–1.84)**	**1.46 (1.14–1.88)**
moderate-to-vigorous intensity physical activity (MVPA) minute/day	0.99 (0.97–1.01)	0.99 (0.98–1.01)	1.00 (0.99–1.02)
Occupational group			
Occupations that require a college education		1.97 (0.36–10.64)	2.03 (0.37–11.17)
Health and service workers and industry		5.31 (0.94–29.87)	**5.90 (1.00–34.67)**
Elementary occupations		**9.18 (1.22–69.34)**	**7.85 (0.98–63.01)**
Managers/professionals		reference	reference
Health status 3 months prior to pregnancy			
Good			1.94 (0.82–4.59)
Poor/not good			2.01 (0.60–7.63)
Excellent			reference
Pelvic girdle pain			
Yes			**5.42 (2.26–12.97)**
No			reference

Note: In model 2–3, the OR are adjusted for working time, age, immigrant background, education and number of children in the household. Missing information: 11 cases miss information on education and 6 cases miss information on pelvic girdle pain

Significant results are shown in bold

After adjusting for self-rated general health and pelvic girdle pain in model 3, sedentary time remained significant (OR 1.46, 95% CI: 1.14–1.88). Pelvic girdle pain was associated with higher odds of having long-term sickness absence in pregnancy (OR 5.42, 95% CI: 2.26–12.97), while self-reported health status 3 months prior to pregnancy was not associated (model 3). There was no significant interaction effect between sedentary time and MVPA (results not shown). Preliminary models including pregnancy-induced emesis (not shown) revealed no change in the association between sedentary hours/day and long-term sickness absence, and emesis was also significantly associated with long term sickness absence. However, since data on emesis was self-reported after the objective recording of MVPA and sedentary time, it was not considered a true confounder and not controlled for in the models.

## Discussion

To the best of our knowledge, the present study is the first to examine the relationship between sedentary time and long-term sickness absence in pregnancy. The present investigation shows that sedentary women have a higher probability than less sedentary women for long-term sickness absence in the 2nd trimester. The association persisted after adjustment for demographic and socioeconomic background variables, health status before pregnancy and in early pregnancy. The odds of long-term sickness absence increased by 46% per daily sedentary hour, after adjustment for confounders and MVPA. Surprisingly, MVPA accumulated in bouts ≥10 min was not associated with long-term sickness absence in pregnancy. We identified no previous studies of the association between sedentary time and long-term sickness absence originating from a sample of pregnant women. In a Dutch intervention in office workers without a control group (52% female), the researchers observed no association between sedentary time and sickness absence over 10 months after the intervention [28]. The contradictory findings may be a result of the use of self-reported sedentary time, which may have introduced measurement error and bias towards the null. In our study, measures of sedentary time were obtained by an objective method less prone to error and bias [40].

Sickness absence could be work- related or pregnancy-related, or a combination of the two. Health problems caused by pregnancy may impair the function required to perform occupational tasks, and inappropriate working conditions may accentuate pregnancy-related health problems. The present results are in line with studies reporting adverse health outcomes associated with sedentary behaviour, suggesting that health outcomes mediate the association between sedentary time and sickness absence. Pelvic girdle pain and low back pain are among the most frequently reported reasons for long-term sickness absence in pregnancy [1, 11] and in the present study we observed that pelvic girdle pain increase the risk of long-term sickness absence in pregnancy. Studies reporting the association between sedentary behaviour and musculoskeletal pain [41] suggest that low back pain and pelvic girdle pain are mediating factors. Another potential mediating factor is fatigue, which is among the frequently reported reasons for sickness absence during pregnancy [1]. While evidence is limited, a positive influence of low-moderate intensity exercises on perceptions of fatigue has been reported [42]. This suggests that prolonged sitting may predispose women for fatigue, and that breaks of sedentary time may improve fatigue. Sedentary behaviour may also influence long-term sickness absence via impaired metabolism, atherosclerosis, insulin resistance, and obesity [26].

The association between sedentary behaviour and sickness absence may also reflect work-related dimensions. Pregnant women who develop pelvic girdle pain have lower tolerance for prolonged sitting [43]. While the estimates for sedentary hours/day in the present study represent the total hours during waking hours, it is likely that the estimates partly reflect occupational sedentary time. Given that women who recorded high volumes of sedentary time are employed in predominantly sedentary occupations, these women have lower tolerance for prolonged sitting at work if they develop pelvic girdle pain during pregnancy. If work is not adjusted, these women may have elevated risk of sickness absence [44].

While previous studies have demonstrated that physically active women have lower risk for sickness absence in pregnancy [27], our results suggest that strategies to reduce sedentary time also might need attention. If the association between sedentary behaviour and sickness absence is causal, there is potential for reduced sickness absence by reducing sedentary time and facilitating breaks to prevent prolonged sitting periods both at work and in leisure time. Thus, whether sickness absence is a result of the adverse health effects of sedentary behaviour or lower tolerance for prolonged occupational sedentary activities, incorporation of work adjustments to break up and reduce sedentary time in prevention programs seems reasonable. Programs to prevent sickness absence, that include initiatives to reduce and break sedentary time, must also be informed by evidence showing that women in occupations that are predominantly walking or standing have higher incidence of sickness absence than those in predominantly sedentary occupations [45]. This may indicate that occupational sedentary time is less important than total sedentary time in explaining sickness absence or that a preventive effect can be expected only in sedentary occupations. We are not aware of experimental studies designed to measure the effect of initiatives to reduce/break sedentary time in pregnant employees on sickness absence. There is a need for research that explores factors that lead to sedentary behavior in pregnancy. Existing studies have focused on individual-level correlates of sedentary behavior; for example, sedentary behavior has been related to personality traits [46] and pregnancy related health problems. Contextual correlates and domain-specific sedentary time have not been addressed [47]. Future interventions should be informed by studies of individual and contextual correlates and determinants of sedentary behaviour.

## Strength and weakness

Strengths of this study include the use of objective data on sedentary behaviour and physical activity in pregnancy and the prospective study design. In addition, the high inclusion rate supports the internal validity, while the population based sample and the heterogeneity of socioeconomic and health related background data supports the external validity. There are several limitations; data on sedentary behavior was not tied to context (i.e. occupational vs leisure), which has been found to be an important distinction in previous research [48]. Data on the context of the sedentary behaviour (i.e. at home, at work, transport) is required to better understand the association with sickness absence. Another limitation is that data on hyperemesis was reported after collection of sedentary behaviour data. Also, data on sickness absence did not include information of the diagnosis, hence, the analysis could not distinguish between work-related and pregnancy-related sickness absence. Analysis of self-reported reasons for sick-leave would provide more insight into potential mediators between sedentary time and sick leave. Although we controlled for self-reported health status prior to pregnancy and pelvic girdle syndrome at visit 1, we cannot be sure that we have captured all relevant health problems predisposing women both to become more sedentary and absent due to sickness. However, the control for self-reported health in model 3 probably attenuates the biasing effect of unmeasured confounders, since self-reported health is correlated with objective measures of health, such as mortality [49] and is considered a valid measure of health status. One more limitation is the crude occupational groups, masking the variation of job demands within groups that may be relevant for understanding sickness absence [8]. Moreover, we did not obtain data on work adjustments which may have been offered to women with musculoskeletal pain.

## Conclusion

We found sedentary behaviour to be strongly related to long-term sickness absence in pregnancy in our cohort. Pregnant women with a sedentary lifestyle have a higher risk of long-term sickness absence from work. The odds of sickness absence increased by 46% for each one hour increase in sedentary time. This may be mediated by health problems due to an inactive lifestyle. Reducing sitting time in pregnancy probably has the potential to improve health, and thereby reduce sickness absence in pregnancy.

## Abbreviations

Cis: 95% confidence intervals.
METs: metabolic equivalents
MVPA: moderate-to-vigorous intensity physical activity
ORs: Odds ratios
SWA: Multi-sensor SenseWear™ Pro3 Armband
